# A phylotranscriptomic dataset of angiosperm species under cold stress

**DOI:** 10.1038/s41597-023-02307-8

**Published:** 2023-06-22

**Authors:** Shuo Wang, Yixian Zhang, Xiaoxue Ye, Yirong Shen, Hua Liu, Xijuan Zhao, Liangyu Guo, Lu Cao, Yunfei Du, Wenwu Wu

**Affiliations:** 1 State Key Laboratory of Subtropical Silviculture, Zhejiang A&F University, Lin’an, 311300 Hangzhou, China; 2 Sanya Research Institute of Chinese Academy of Tropical Agricultural Sciences, Yumin Road 7, Sanya, 572025 China

**Keywords:** Abiotic, Plant evolution

## Abstract

Angiosperms are one of the most diverse and abundant plant groups that are widely distributed on Earth, from tropical to temperate and polar zones. The wide distribution of angiosperms may be attributed to the evolution of sophisticated mechanisms of environmental adaptability, including cold tolerance. Since the development of high-throughput sequencing, transcriptome has been widely utilized to gain insights into the molecular mechanisms of plants in response to cold stress. However, previous studies generally focused on single or two species, and comparative transcriptome analyses for multispecies responding to cold stress were limited. In this study, we selected 11 representative angiosperm species, performed phylotranscriptome experiments at four time points before and after cold stress, and presented a profile of cold-induced transcriptome changes in angiosperms. Our multispecies cold-responsive RNA-seq datasets provide valuable references for exploring conserved and evolutionary mechanisms of angiosperms in adaptation to cold stress.

## Background & Summary

Due to their sessile nature, plants have evolved sophisticated signaling pathways in adaptation to complex environmental fluctuations such as extreme temperature, high salinity, and drought^1,2^. Low temperature is one of the environmental stresses that causes a series of physiological and metabolic changes in plants^3,4^, and severe low temperature even causes plant death, which seriously threatens food and seed security. However, plants are not completely as passive as they seem. Generally, temperate plants could increase their freezing tolerance by preexposure to low but non-freezing temperature environment for a few days or weeks, the process is known as cold acclimation^5^. Cold acclimation is a complex process that involves multiple physiological and biochemical changes, such as modification of lipid composition, changes of protein and carbohydrate composition, and accumulation of anti-freezing and anti-oxidative substances^5^. Accumulating evidence demonstrates that most of these changes are mainly due to the expression of cold-responsive (*COR*) genes, which are induced by cold acclimation and play important roles in plants resistance to low temperatures^6–8^. Exploring the regulatory mechanisms of *COR* genes in cold acclimation contributes to our understanding of plants adapting to extremely low temperatures and molecular breeding.

Since the development of high-throughput sequencing, RNA sequencing (RNA-seq) has been widely utilized to gain insights into the molecular mechanisms of plants in response to cold stress. To date, molecular responses of cold stress have been widely investigated and characterized in many plant species, such as *Arabidopsis thaliana*^9^, *Oryza sativa*^10^, *Zea mays*^11^, *Ocimum americanum*^12^, *Malus sieversii*^13^, *Nicotiana tabacum*^14^, *Phyllostachys edulis*^15^, *Betula platyphylla*^16^, and *Populus trichocarpa*^17,18^. However, these prior studies generally focus on single or two species, and comparative transcriptome analyses for multispecies responding to cold stress were less investigated.

As the most diverse and abundant plant groups, angiosperms are widely distributed on Earth from tropical to polar terrestrial zones. The wide distribution of angiosperms may be attributed to the evolution of advanced and sophisticated mechanisms of environmental adaptability^19^. In this study, we present cold-stress transcriptome analyses of 11 angiosperm species, including six eudicots (*Arabidopsis thaliana*, *Betula pendula*, *Populus trichocarpa*, *Carya illinoinensis*, *Glycine max*, and *Cucumis sativus*) and five monocots (*Oryza sativa*, *Setaria italica*, *Hordeum vulgare*, *Zea mays*, and *Phyllostachys edulis*). For each of the species, seedlings were treated at four different time points of cold stress (0, 2, 24, and 168 hours (h)), and three biological replicates were performed for each time point. Finally, we generated a total of 132 RNA-seq datasets from the 11 angiosperms under cold treatments. Our multispecies cold-responsive RNA-seq datasets provide valuable references for exploring the conservation and evolution of cold-responsive molecular mechanisms of angiosperms.

## Methods

### Plant materials and growth conditions

**Fig. 1:**
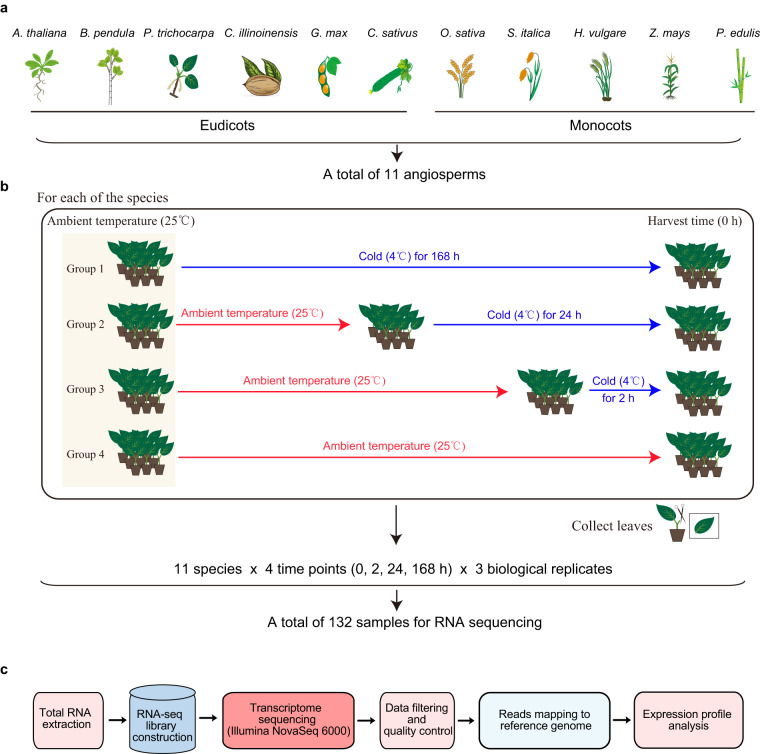
Overview of multispecies cold treatment experiment and transcriptome analysis pipeline. (**a**) A total of 11 representative angiosperm species were selected for generating cold stress RNA-seq dataset. The selected species include six eudicots: *Arabidopsis thaliana* (*A. thaliana*), *Betula pendula* (*B. pendula*), *Populus trichocarpa* (*P. trichocarpa*), *Carya illinoinensis* (*C. illinoinensis*), *Glycine max* (*G. max*), and *Cucumis sativus* (*C. sativus*), and five monocots: *Oryza sativa* (*O. sativa*), *Setaria italica* (*S. italica*), *Hordeum vulgare* (*H. vulgare*), *Zea mays* (*Z. mays*), and *Phyllostachys edulis* (*P. edulis*). (**b**) Cold stress treatments of the 11 selected species. Different time points (0, 2, 24, and 168 h) of cold stress (4 °C) treatments were separately performed in each of the species. Leaves of the treated seedlings were collected, and three biological replicates were performed for each cold treatment. Finally, we obtained 132 samples of multispecies under cold stress treatments (see Methods). (**c**) A brief pipeline of RNA-seq experiment and analysis. The total RNA of the samples was extracted for Poly (A) RNA enrichment and cDNA library construction. The cDNA libraries were sequenced on Illumina NovaSeq 6000. After data filtering and quality control, the clean reads of the transcriptomes were mapped to their reference genomes, and the expression profiles of cold-responsive genes were analyzed (see Methods).

Seedlings of 11 selected representative angiosperm species, including six eudicots (*A. thaliana*, *B. pendula*, *P. trichocarpa*, *C. illinoinensis*, *G. max*, and *C. sativus*) and five monocots (*O. sativa*, *S. italica*, *H. vulgare*, *Z. mays*, and *P. edulis*) (Fig. 1a), were cultured in an artificial climate chamber with 25 °C at a photoperiod of 16/8 h light/dark cycle. For *A. thaliana*, three-week-old seedlings were prepared for utilization in cold treatment. For other species, young seedlings growing up to ~30 cm in height were prepared.

### Cold stress treatment

Under cold stress, gene expression is reprogrammed to form a hierarchical regulatory network, which is constituted of rapid, early, and late cold-responsive genes. To obtain these genes, we performed cold stress treatments of the 11 selected representative angiosperm species under different time points (0, 2, 24, and 168 h). For each species, seedlings with relatively uniform growth and physiological state were selected and divided into four groups (Group 1 to Group 4). To ensure that the four seedling groups of cold treatments (0, 2, 24, and 168 h) could be harvested in the same development stage at the same time on a day, the seedling group of cold treatment for 168 h was first cultured in the artificial climate chamber with 4 °C a week (168 h) before harvest, and then were seedling groups of cold treatment, respectively, for 24, 2, and 0 h at proper times. After cold stress treatments, we collected the fourth expanded leaves of the treated seedlings, which are generally considered as mature healthy leaves at similar developmental stages. For each cold treatment, three biological replicates were performed (Fig. 1b).

### RNA extraction, library construction, and sequencing

The total RNA of the collected leaves from each species was isolated and purified using TRIzol reagent (Invitrogen, Carlsbad, CA, USA) following the manufacturer’s procedure. The RNA amount and purity of each sample were assessed by NanoDrop ND-1000 (NanoDrop Technologies, Wilmington, DE, USA) and Agilent 2100 Bioanalyzer (Agilent Technologies, Palo Alto, California, USA). Poly (A) RNA was purified from total RNA using poly-T oligo-attached magnetic beads to generate strand-specific cDNA libraries containing inserts of approximately 150–200 bp in size. In total, 132 cDNA libraries from 11 species under four time points before and after cold stress (0, 2, 24, and 168 h) were constructed for transcriptome analysis. The libraries were sequenced on Illumina NovaSeq 6000 sequencing system (2 × 150 bp paired-end reads) at LC-Bio Technology CO., Ltd. (Hangzhou, China) according to the manufacturer’s instructions (Fig. 1c).

## Data Records

The above 132 RNA-seq datasets have been deposited into the NCBI BioProject with the accession number PRJNA767196^20^. The read-count data matrix of cold-treated samples in each of the 11 species is available at figshare data repository (10.6084/m9.figshare.22643245.v1)^21^. DEGs between different time points of cold treatments and normal conditions for each species time points are also available at figshare (10.6084/m9.figshare.22643074.v1)^22^.

## Technical Validation

### Data filtering and quality control

The raw data in fastq format were processed by Trimmomatic v0.39^23^ to remove the Illumina adapter contamination and low-quality bases. After filtering, quality control of the clean reads in each RNA-seq dataset was assessed using FastQC^24^ (https://www.bioinformatics.babraham.ac.uk/projects/fastqc/). Q20 and Q30 average values of the 132 libraries were 99.84% and 98.29%, respectively (Dataset 1). The average GC content of the dataset was 47.84% (Dataset 1). Overall results of filtering and quality of clean reads indicated that the sequencing progressed adequately composing a series of high-quality RNA-seq datasets.

### Reference genomes assessment and reads mapping

**Fig. 2:**
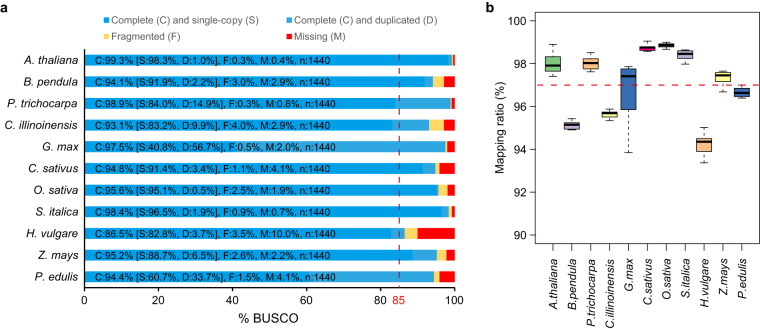
Genome annotation quality and reads mapping of RNA-seq data to reference genomes. (**a**) Benchmarking Universal Single-Copy Orthologs (BUSCO) scores of the 11 reference genomes. (**b**) Boxplots showing read mapping ratios of RNA-seq data to the reference genomes. In each species, a total of 12 RNA-seq samples were obtained according to four time points, each with three biological replicates. The dashed line indicates the average read mapping ratio of the 132 RNA-seq samples.

Genome sequences of 11 angiosperms were downloaded from public databases. In detail, genome sequences of *A. thaliana* were downloaded from TAIR10^25^, *B. pendula* and *C. sativus* were downloaded from NCBI^26^, *P. trichocarpa*, *G. max*, *O. sativa* and *S. italic* were downloaded from Phytozome v13.1^27^, *H. vulgare* and *Z. mays* were downloaded from Ensembl Plants^28^, *C. illinoinensis* and *P. edulis* were downloaded from GigaDB^29^. We first used BUSCO v.3.0.2^30^ to detect the quality of the reference genomes. The complete BUSCO values of the 11 reference genomes ranged from 86.3% to 99.3%, among which 10 genomes were over 90% (Fig. 2a), indicating that the reference genomes were appropriate and of high quality. To detect the mapping ratio of the transcriptomes, clean reads from each sample were mapped to their corresponding reference genome by HISAT2 v2.1.0^31^. The average mapping ratio of the RNA-seq samples in each species ranged from 94.23% to 98.84% (Dataset 2 and Fig. 2b).

### Principal component analysis

**Fig. 3:**
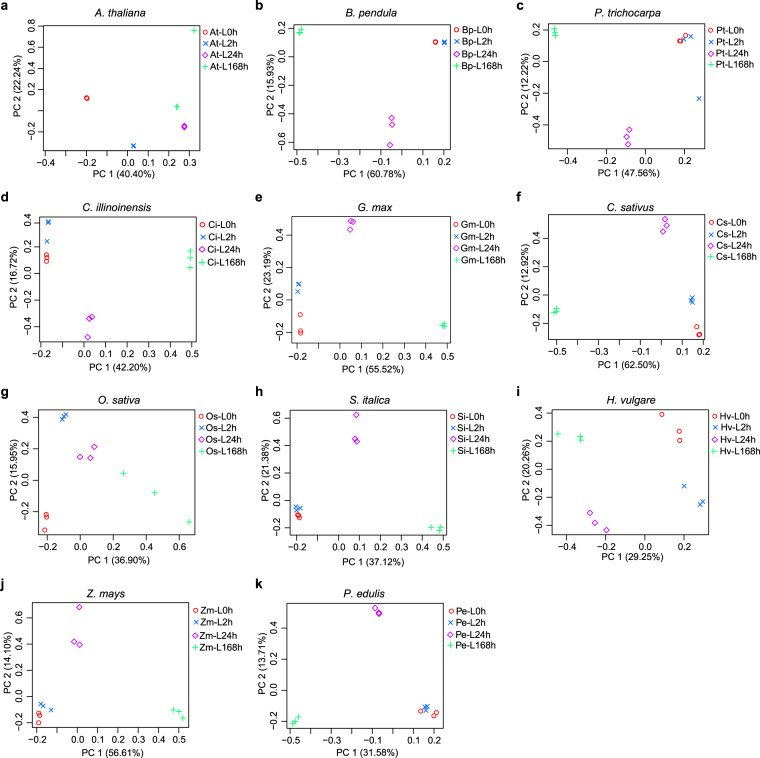
Principal component analysis (PCA) across RNA-seq samples of cold treatments in each of the species. PCA was performed by the script of PtR in Trinity^33^.

StringTie v2.0.3^32^ was further utilized to estimate the gene expression level (trimmed mean of M value, TMM) based on all mapped reads to the reference genome, and the expressed genes in each sample were identified with the average TMM > 0.5 across three replicates of the sample (Dataset 3). Read-count data can be available at Figshare (10.6084/m9.figshare.22643245.v1)^21^. Using the script of PtR in Trinity^33^, we performed principal component analysis (PCA) based on the mapped count read table of the RNA-seq samples for each species. PCA results showed a clear clustering according to the same time point of cold treatments (Fig. 3), suggesting a high consistency of the biological replicates.

### Identification of differentially expressed genes

**Fig. 4:**
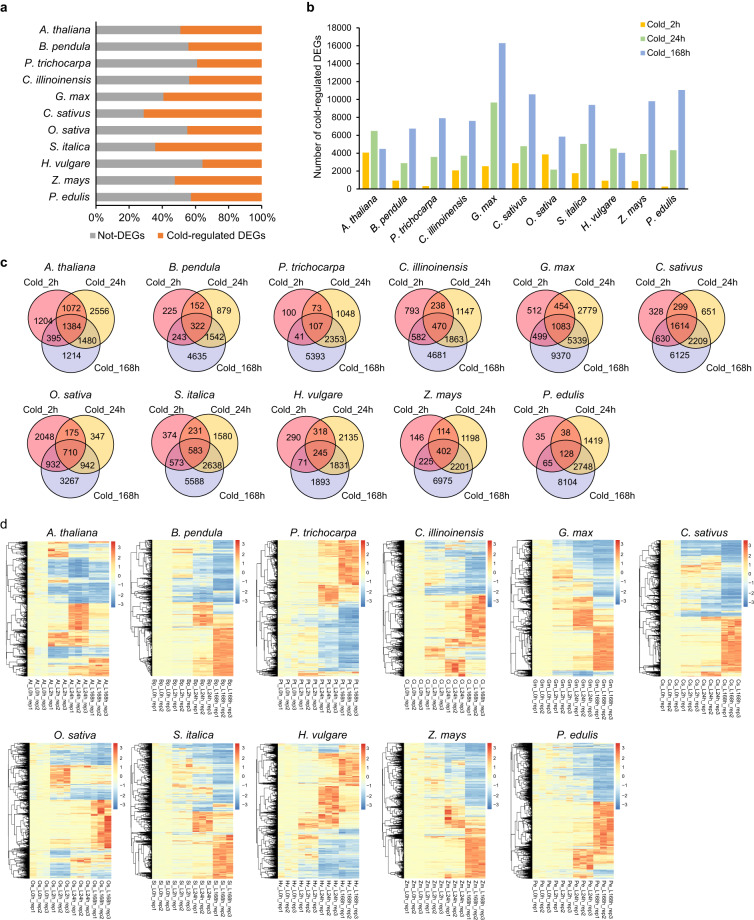
Expression profiles of cold-responsive genes in the 11 selected species. (**a**) Percentages of DEGs in the expressed genes in each species. (**b**) The number of DEGs in each of the species between cold treatments (2, 24, and 168 h) and normal condition (0 h). (**c**) Venn diagrams showing unique and overlapping DEGs from different time points of cold treatments in each species. (**d**) Heatmaps of the DEGs among different time points of cold treatments in each species.

Differentially expressed genes (DEGs) between two time points before and after cold treatments (0 h versus 2, 24, or 168 h) were obtained using edgeR^34^, DESeq 2^35^, and Ballgown^36^. In brief, analyzed from at least two of the three methods, those genes with a mean TMM ≥ 1 across the compared samples that had an adjusted *P*-value or false discovery rate (FDR) <0.05 and an absolute value of fold change ≥ 2 were considered to be DEGs. For each species, the number of DEGs under different time points of cold treatments (2, 24, and 168 h) compared with the control (0 h) was calculated (Fig. 4a,b, Dataset 4). The meta-data of DEGs are available under Figshare DEG tables of 11 angiosperm species under different time points of cold treatments^22^. Venn diagrams of the DEGs obtained from different time points of cold treatments were analyzed (Fig. 4c). Additionally, we used R programming to plot heatmaps of the expression of DEGs in each species (Fig. 4d, Dataset 5).

## Supplementary information

Dataset 1

Dataset 2

Dataset 3

Dataset 4

Dataset 5

## Data Availability

Software and their versions used for RNA-seq analysis were described in Methods. No custom code was used to generate or process the data described in the manuscript.
